# Effects of Dust Exposure on the Respiratory Health Symptoms and Pulmonary Functions of Street Sweepers

**DOI:** 10.21315/mjms2018.25.6.8

**Published:** 2018-12-28

**Authors:** Raheleh Hashemi Habybabady, Hannaneh Nasibi Sis, Fatemeh Paridokht, Fatemeh Ramrudinasab, Ali Behmadi, Bentolhoda Khosravi, Mahdi Mohammadi

**Affiliations:** 1 Health Promotion Research Centre, Department of Occupational Health Engineering, Zahedan University of Medical Sciences, Zahedan, Iran; 2 BSc student in Occupational Health Engineering, Zahedan University of Medical Sciences, Zahedan, Iran; 3 Health Promotion Research Centre, Department of Biostatistics & Epidemiology, Zahedan University of Medical Sciences, Zahedan, Iran

**Keywords:** pulmonary function, dust, spirometry, respiratory symptoms, exposure

## Abstract

**Background:**

The most common risks for street sweepers are respiratory symptom and airway obstruction increases as a result of dust inhalation. The aim of this study was to compare the respiratory symptoms and pulmonary functions of dust-exposed street sweepers with those of unexposed individuals.

**Methods:**

This was a retrospective cohort study of 84 street sweepers with occupational dust exposure (exposed group) and 80 office workers (control group) working for the municipality of Zahedan in Iran. Each of the participants completed the American Thoracic Society respiratory questionnaire, and their lung functions were measured using a calibrated spirometer.

**Results:**

The respiratory symptom frequencies were significantly higher in the exposed group than in the reference group (*P* < 0.05). Specifically, coughing was the most common complaint of the street sweepers (81%) when compared to the controls (16.3%). The means of the peak expiratory flow and forced expiratory flow at 25%–75% of the pulmonary volume were significantly less in the exposed group than in the control group.

**Conclusion:**

Occupational exposure and unhealthy working conditions are the most likely causes of mild obstructive disease and pulmonary function parameter changes. Providing street sweepers with the appropriate respiratory protection equipment, as well as periodic spirometry for the early diagnosis of pulmonary dysfunction, could be effective for preventing many types of pulmonary damage.

## Introduction

Street sweepers play important roles in environmental health by maintaining the cleanliness of the streets; however, these individuals are exposed to many risks. Unfortunately, the socioeconomic status and educational levels of street sweepers are low, and less attention is paid to their health (1, 2). Dust includes the most commonly found harmful particles in the atmosphere, and street sweepers are exposed to a combination of soil, sand and gravel dust particles, vehicle dust, bioaerosols and plant particles (3). During pulmonary ventilation, tiny particles are deposited in the lower parts of the respiratory system, and they become inaccessible to the self-cleansing mechanisms of the body, such as mucociliary clearance (4, 5). The inhalation of external materials triggers the lungs to react in different ways, including airway irritation, asthma exacerbation, inflammatory reactions and fibrosis. While short-term exposure to dust may cause immediate and severe damage, chronic or persistent exposure for months or years may result in permanent illnesses or injuries. In some studies, sneezing, coughing, eye irritation, lung tissue swelling, asthma and throat infections were found to be more prevalent among individuals exposed to occupational dust. Moreover, the symptoms associated with impaired lung function may lead to occupational lung diseases (6, 7).

Pulmonary diseases due to occupational exposure are mostly related to dust inhalation and the deposition of inhaled particles, depending on the size, physical properties and chemical properties of the aerosol, frequency and duration of exposure, and individual response to dust particles in the lungs (8). The available evidence corroborates the relationships between the exposure to dust produced by traffic and respiratory disorders, reduced pulmonary function, cardiovascular disease and lung cancer (9).

Chronic obstructive pulmonary disease (COPD) is the third leading cause of death in the United States (10). The incidence and COPD-related mortality has been higher in women than men in recent years (11). The long and continuous inhalation of non-industrial dust in Pakistani street sweepers has been reported to be one of the critical factors in the development of COPD, resulting in obstructive ventilatory patterns (12). When compared with other health hazards, respiratory problems are more prevalent among Indian cleaners (13). Moreover, the effects of occupational dust exposure on the reduction of pulmonary function has been confirmed (14–16). The lung diseases seen in street sweepers are often due to the deposition of harmful dust particles that are inhaled while sweeping. Because these tissues are not damaged during short-term dust exposure, street sweepers with constant dust exposure first develop pulmonary obstruction, which leads to restrictive ventilatory defects. However, the early diagnosis of respiratory disease helps to block the disease progression (5).

Air pollution is one of the most important environmental challenges in Iran, and it becomes more complicated in desert areas, like Zahedan in southeastern Iran, which has an average of 70 dusty days per year (17). According to the spatial and temporal analyses of dust storms in Iran, the greatest number of dusty days in the country occur in Zabol, which is located 250 km away from Zahedan to the north, in the Sistan and Baluchistan province. After Zabol, the most dusty days have been recorded in Zahedan, Bushehr, Tabas, Bandar Abbas, Jask, Iranshahr, Hamedan and Ahwaz (18). Based on the Environmental Protection Agency standards, the limit is a PM_10_ concentration of 150 μg/m^3^ once a year, at the most. However, in the city of Zahedan, amounts exceeding this concentration often occur (189 μg/m^3^ on average), especially in the summer, with seasonal winds (called ‘wind of 120 days’) in the northern part and monsoon systems in the southern part of the province (19).

Although the effects of inhaling dust are known, no studies have been conducted on the lung function of street sweepers working for the Zahedan municipality. The excessive exposure to dust in this city may provide useful information for further research. Moreover, given the large number of workers employed by the city services, the need to conduct research exploring the health status of this hard-working population becomes even more pressing. Therefore, this study was performed to evaluate the pulmonary functions and respiratory health symptoms of street sweepers and compare them to a control group. Additionally, the relationships between the dust exposure durations and the lung function parameters in the street sweepers of the Zahedan municipality were also evaluated.

## Methods and Subjects

### Study Design

This retrospective cohort study was carried out among the street sweepers working in the Zahedan municipality. Street sweepers with more than 5 years of work experience were included in this research. Individuals with histories of asthma, COPD, tuberculosis, acute and chronic respiratory infections, abdominal or thoracic surgeries, cardiovascular diseases, diabetes and hypertension, and those with histories of working in other occupations were excluded from the study.

### Sampling

Out of all the street sweepers working for the Zahedan municipality, 84 individuals were selected via simple random sampling. These sweepers worked 8 h each day, 6 days a week, without using respiratory protective equipment. In addition to the exposure group, 80 employees with no occupational exposure to respiratory pollutants were randomly chosen from the office workers in the municipality as the control group.

The sample size was calculated based on the results of a pilot study using the same methodology in the same population, including 25 street sweepers and 25 controls. According to the formula for comparing the two means, with a type I error of 0.05 and power of 95%, the largest sample size was calculated for the forced vital capacity (FVC), with a means [standard deviation (SD)] of 93.5 (15.1) in the unexposed group and 84.8 (16.5) in the exposed group. Therefore, 86 cases were chosen for each group based on a simple random sampling method. There were two drop outs in the exposed group and six drop outs in the unexposed group due to a lack of participant cooperation.

### Demographic Information

The demographic information was collected from the street sweepers and the control subjects as follows: sex, age, weight, height, work experience, educational level, marital status and smoking. Those individuals who had smoked one or more cigarettes each day over the past 30 days were considered to be cigarette smokers, and those who had consumed 100 cigarettes or less during their lifetime and had stopped smoking for at least 30 days were considered to be former cigarette smokers (20).

### Respiratory Disorder Symptoms

All of the participants completed the American Thoracic Society respiratory questionnaire (21). This scale assesses the respiratory state of individuals in terms of their signs and symptoms, such as coughing, phlegm, wheezing and dyspnoea, while taking into account their smoking, work experience and medical histories. The data obtained from this questionnaire was used to determine the prevalence of respiratory symptoms between the two groups.

### Pulmonary Function Test

Pulmonary function tests (PFTs) were performed in both groups based on the standard instructions (22) using a calibrated spirometer (Spirobank II Basic; MIR Medical International Research, Rome, Italy) with an accuracy of 3% and a flow of 5%. The actual parameters that were measured included the FVC, forced expiratory volume in the first second (FEV_1_), FEV_1_/FVC ratio, peak expiratory flow (PEF) and forced expiratory flow at 25%–75% of the pulmonary volume (FEF_25%–75%_). The spirometer also calculated the predicted pulmonary function parameters based on the age, height, weight, sex, smoking and race (23). Each of the participants was advised to refrain from eating and smoking for 2 h before the test, avoid heavy exercise, and wear comfortable clothes (tight clothes restrict thoracic movement). Additionally, all of the participants were provided with proper training, and their weights and heights were measured while in the standing position. The body mass index (BMI) was calculated, and after a complete rest period, each subject was asked to sit and apply the nose clips for the test. The test was carried out three times for each subject, and the maximum value of the consecutive tests was recorded. Those individuals who did not perform the manoeuvres in a satisfactory manner after three tries were excluded from the study. The ratio of the actual values to the predicted values for the pulmonary function parameters was considered for the data analysis.

### Statistical Analysis

The data were analysed using the Statistical Package for the Social Sciences version 16 (SPSS Inc., Chicago, IL, USA). The quantitative and qualitative variables were described as the means (SD) and frequency (percentage), respectively. The normal distribution of the quantitative variables was confirmed by the Shapiro-Wilk test. In order to compare the street sweepers and the control group in terms of the quantitative demographic variables, an independent samples *t*-test was used. For the pulmonary function parameter comparisons, an analysis of covariance was used by adjusting it for the BMI and work history. Finally, a likelihood ratio test was employed to assess the odds ratio of the street sweepers experiencing respiratory symptoms when compared to the controls.

## Results

In this study, 84 street sweepers exposed to dust and 80 office workers in the Zahedan municipality were investigated. The ages of the participants ranged from 23 years old to 61 years old and 21 years old to 78 years old in the exposed and control groups, respectively. The work experiences varied from 5 years to 30 years in the exposed group and 5 years to 41 years in the unexposed group. Furthermore, 31.3% and 17.5% of the individuals in the unexposed group and 14.3% and 6% of the individuals in the exposed group were overweight and obese, respectively.

There was a significant difference between the two groups in terms of the BMI (*P* < 0.001) and work history (*P* < 0.001). The means and SDs of some of the demographic characteristics are shown in Table 1.

Current cigarette smokers made up 8.8% of the unexposed group and 11.9% of the exposed group (Table 2). However, there was no statistically significant difference between the two groups. The smoking duration means were 16.2 (SD = 15.9) for the former cigarette smokers and 14.8 (SD = 12.9) for the current cigarette smokers.

Table 3 provides a comparison between the respiratory symptoms in the exposed and unexposed groups. All of the respiratory symptoms, including coughing, phlegm, coughing with phlegm, dyspnoea and wheezing, were more prevalent in the street sweepers than in the office workers (*P* < 0.001). The chances of experiencing coughing, phlegm, dyspnoea and wheezing symptoms were 21.9, 48.6, 4.3 and 15.8 times higher in the exposed group than in the unexposed group.

Among the pulmonary function parameters, the means of the PEF and FEF_25%–75%_ of the street sweepers were significantly less than those of the controls (*P* < 0.001). Although the means of the FEV_1_ and FEV_1_/FVC parameters were not significantly different between the two groups, the means differences were considerable. Moreover, there was no significant difference between the street sweepers and the controls in terms of the FVC (Table 4).

## Discussion

In this study, the BMI means for the unexposed group (25.8 kg/m^2^) was significantly higher than that for the exposed group (22.4 kg/m^2^). This could be due to the greater physical activity of the exposed individuals with regard to their duties, such as sweeping, bending and straightening while walking in the streets, when compared to the sedentary office workers in the same municipality.

Based the results of this study, the respiratory symptoms, including coughing, phlegm, coughing with phlegm, dyspnoea and wheezing, were significantly more common in the street sweepers than in the controls. Coughing and wheezing were five and six times more common, respectively, in the exposed group than in the control group. Therefore, it can be inferred that the street sweepers’ exposure to dust increased their chances of developing respiratory symptoms. In the current study, the chances of experiencing coughing and phlegm were 21.9 and 48.6 times greater in the sweepers than in the controls, respectively. Similarly, Neghab et al. (24) reported a higher prevalence of respiratory symptoms in the sweepers when compared to the control group. Gholamie et al. (25) found that coughing was the most common respiratory complaint (43%) caused by exposure to high amounts of dust and gas from fossil fuels. Two studies conducted in Egypt reported coughing to be approximately three times more common in the street sweepers than in the controls (26, 27). The direct exposure of street sweepers to organic and mineral dust from sweeping and the lack of proper respiratory protection equipment can lead to respiratory symptoms in this population. Dust particles, smoke and other biological substances irritate the respiratory system, causing coughing and other respiratory complications. Some other studies have reported similar relationships between respiratory symptoms and carbon dust (23), coffee dust (28) and cement dust (29). In contrast, Bünger et al. (30) did not find any significant difference between the incidence of respiratory symptoms in the control group and in the street sweepers. In Greece, in a study of 104 street sweepers who worked 6 h a day, 5 days a week, coughing with phlegm was not significantly higher in the street sweepers than in the control group. The latter could be due to the Greek Committee for Occupational Health and Safety laws requiring that street sweepers with significant respiratory symptoms be transferred to other municipal departments. If their respiratory problems lead to disability, that person is considered to be disabled (31).

In the current study, the means of the pulmonary functions (FEV_1_, PEF and FEF_25%–75%_) in the exposed group were significantly lower than those in the control group. Although the FVC values were lower in the exposed group, the difference was not significant, implying that the dust had not yet caused severe damage to the small airways of lung. The FEV_1_/FVC ratio of the exposed group [98.0] was nearly significantly different (*P* = 0.052) from that of the control group [103.7]. When considering the fact that both the FEV_1_ value and FVC value exceeded 80% in the exposed group and that the FEV_1_/FVC ratio was above 75%, it can be concluded that there was no evidence of obstructive or restrictive lung diseases in the exposed group. Nevertheless, because the FVC value was normal and the FEF_25%–75%_ value was less than 75% in the exposed group (revealing a significant difference from the control group), the street sweepers could have developed mild obstructive pulmonary disease. This condition affects most of the small airways, and it is likely to aggravate as the work experience increases (32).

The current study results are in line with those from a study of workers in a calcium hypochlorite and alkali chlorine unit in the petrochemical industry (23) and another study of street sweepers (24) that reported significant differences between the FEV_1_/FVC ratios and FEV_1_ values between the exposed and control groups. In contrast, two studies in India reported different results. Johncy et al. (4) found that all of the pulmonary function parameters, including the FVC, FEV_1_, PEF and FEF_25%–75%_, were significantly lower in the exposed group than in the control group, while the FEV_1_/FVC ratios of the two groups did not reveal a significant difference. Ajay et al. (3) reported that the FVC values of 50 female street sweepers were significantly lower than those of the 50 women in the control group who had no occupational exposure. The reason for the difference between the current study and the two latter studies in India may be related to the dissimilarities in the dust concentrations, sexes of the exposed individuals, anatomical and physiological differences between males and females, differences in the pulmonary functions of the individuals, and the specific occupational conditions and races of the study subjects. Because the pulmonary function capacity of women is less than that of men (32), it is likely that women’s sensitivity to dust inhalation is greater than that of men. Additionally, women also display a higher degree of bronchial response due to hormone fluctuations after puberty (33). In one previous study, a comparison of the pulmonary functions of male and female cigarette smokers showed that female lungs were relatively smaller than male lungs, and the metabolism of the compounds in the cigarette smoke differed between the genders (11). Therefore, the lower pulmonary function parameter levels in women make them more vulnerable when they are exposed to pollutants. Contrary to the results of the current study, another study of 52 male street sweepers in Switzerland showed that there were no significant differences in the pulmonary function parameters between the street sweepers and the control group (34), which could have been due to the smaller sample size and remarkably better working conditions in that study.

In the present study, there was no correlation between the work experience and pulmonary functions of the exposed group, which is consistent with the findings of the study by Neghab et al. (35). Conversely, Mariammal et al. (36) and Anwar et al. (12) suggested that the number of years of employment affected the pulmonary parameters. Perhaps the lack of significance observed in the current study may be due to the differences in the factors, including the exposure duration, dust concentration and particle size, previous occupations, and the absence of accurate work experience documentation of Zahedan street cleaners. Moreover, considering the relatively young age of the participants evaluated in this study, with a means age of 37.4 (SD = 7.6) years old, it is possible that the body’s defence systems are still able to regulate pulmonary function. It should be noted that a number of the street sweepers used special scarves, which are common among the local Baloch ethnic group, as respiratory masks to reduce the flow of dust into their lungs.

Because the work experience and BMI means were greater in the control group than in the exposed group, the pulmonary function reduction was mainly due to the dust exposure and working conditions. Neither group had histories of respiratory illness or dust exposure prior to employment, and none of them had second jobs; therefore, one may assume that the changes in the pulmonary function parameters must have originated from the street sweepers’ occupational exposure.

The cigarette smoking duration and number of smokers were not significantly different between the two groups in the current study. Similarly, other studies have reported no relationships between cigarette smoking and pulmonary function (24, 34). Contrary to the aforementioned results, Shadab et al. (5) assessed the pulmonary functions of 110 street sweepers with working histories of more than 5 years. They demonstrated that dust exposure caused obstructive pulmonary dysfunction among the street sweepers, a condition which is exacerbated by smoking. The reason for this inconsistency in the findings could be the lower number of smokers in the present study when compared to that in the latter study (10 versus 20 individuals, respectively).

## Conclusion

Although the results of this study did not establish the temporal relationships of the dust risk, they did indicate that the pulmonary function parameter changes of the street cleaners were associated with their occupational exposure and working conditions. As a result, the frequency of respiratory symptoms among the street sweepers was greater than that of the control group. In order to prevent respiratory disorders in this population, and reduce dust while sweeping, it is strongly recommended that preventive measures, such as brooms with large handles, modern cleansing equipment, sprinkling water on the street before sweeping, limiting the duration of work to 3 or 4 days a week, and using appropriate respiratory protection, be introduced by municipal authorities. Furthermore, a periodic assessment of lung function needs to be conducted via spirometry in order to diagnose pulmonary dysfunction early in this financially deprived population. Eventually, if possible, individuals with respiratory problems or significant pulmonary function parameter reductions should be transferred to other municipal departments.

## Figures and Tables

**Table 1:** Means (standard deviations) of the demographic characteristics of the exposed and unexposed groups

Demographic characteristics	Unexposed (*n* = 80)	Exposed (*n* = 84)	*t*-statistic (df)	*P*-value^a^

	Mean (SD)	Mean (SD)		
Age (years)	42.1 (10.9)	37.4 (7.6)	3.20 (139.96)	0.002
Weight (kg)	75.6 (14.1)	64.4 (12.9)	5.31 (162.00)	< 0.001
Height (cm)	170.8 (7.7)	169.7 (6.4)	0.99 (153.92)	0.323
BMI (kg/m^2^)	25.8 (4.2)	22.4 (4.6)	4.98 (162.00)	< 0.001
Work history (years)	18.2 (9.8)	11.5 (5.4)	5.34 (122.12)	< 0.001

BMI = body mass index.

a Independent *t*-test

**Table 2:** The frequency distributions of cigarette smoking in the exposed and unexposed groups

Group Smoking	Unexposed (*n* = 80)	Exposed (*n* = 84)	Total *n* (%)

	*n* (%)	*n* (%)	
Never smoker	70 (87.5)	71 (84.5)	141 (86.0)
Former smoker	3 (3.8)	3 (3.6)	6 (3.7)
Convent smoker	7 (8.8)	10 (11.9)	17 (10.4)

**Table 3:** Respiratory symptoms associated with dust exposure

Respiratory symptoms		Unexposed (*n* = 80)	Exposed (*n* = 84)	OR (95% CI)	*P*-value^a^

		*n* (%)	*n* (%)		
Cough	Yes vs No^b^	13 (16.3)	68 (81.0)	21.9 (9.8, 49)	< 0.001
Phlegm	Yes vs No^b^	1 (1.3)	32 (38.1)	48.6 (6.4, 366.8)	< 0.001
Cough with phlegm	Yes vs No^b^	0 (0.0)	16 (19.0)	–	< 0.001
Dyspnea	Yes vs No^b^	22 (27.5)	52 (61.9)	4.3 (2.2, 8.3)	< 0.001
Wheezing	Yes vs No^b^	9 (11.3)	56 (66.7)	15.8 (6.9, 8.3)	< 0.001

OR = odds ratio, CI = confidence interval,

a likelihood ratio test,

b the reference category

**Table 4:** Comparison of the pulmonary function parameters of the exposed and unexposed groups

Pulmonary function parameters	Unexposed (*n* = 80)	Exposed (*n* = 84)	*P*-value^a^	Adjusted estimated mean^b^	
	
	Mean (SD)	Mean (SD)		Unexposed	Exposed
FVC	93.1 (13.9)	89.6 (17.6)	0.434	92.5	90.2
FEV_1_	92.7 (14.3)	84.3 (19.2)	0.052	91.4	85.5
FEV_1_/FVC (%)	103.7 (6.8)	98 (14.8)	0.074	102.7	98.9
PEF	88.8 (19.5)	69.5 (24.8)	< 0.001	86.9	71.3
FEF_25%–75%_	89.8 (21.1)	70.9 (24.5)	< 0.001	88.1	72.5

a Analysis of covariance,

b adjusted mean for BMI and work history
